# Supplementary material for: Transferrin Saturation/Hepcidin Ratio Discriminates TMPRSS6-Related Iron Refractory Iron Deficiency Anemia from Patients with Multi-Causal Iron Deficiency Anemia
Source: Int J Mol Sci. 2022 Feb 8;23(3):1917. doi: 10.3390/ijms23031917 (PMC8836508; doi:10.3390/ijms23031917)
Supplement: Supplementary file 1 [file ijms-23-01917-s001.zip › ijms-1567354-supplementary.pdf]

Supplementary Materials

Table S1. Performance of the TSAT/hepcidin ratio in distinguishing IRIDA patients from IDA controls in absence of inflammation and without recent iron therapy.

	**IRIDA patients (n = 20) versus IDA controls (n = 39)**						
**TSAT/hepcidin ratio**	AUC	95% CI of AUC	Cut-off value	Sensitivity	95% CI of Sensitivity	Specificity	95% CI of Specificity
A. Hepcidin <0.5 nM = 0.25 nM							
Total IRIDA group versus IDA controls	1.000	1.000-1.000	<5.6%/nM	100%	84-100%	100%	91-100%
Biallelic IRIDA patients versus IDA controls	1.000	1.000-1.000	<4.3%/nM	100%	74-100%	100%	91-100%
Monoallelic IRIDA patients versus IDA controls	1.000	1.000-1.000	<5.6%/nM	100%	70-100%	100%	91-100%
B. Hepcidin <0.5 nM = 0.49 nM							
Total IRIDA group versus IDA controls	1.000	1.000-1.000	<3.9%/nM	100%	84-100%	100%	91-100%
Biallelic IRIDA patients versus IDA controls	1.000	1.000-1.000	<2.6%/nM	100%	74-100%	100%	91-100%
Monoallelic IRIDA patients versus IDA controls	1.000	1.000-1.000	<3.9%/nM	100%	70-100%	100%	91-100%
C. Exclusion of IDA control with a monoallelic pathogenic TMPRSS6 variant without IRIDA phenotype	1.000	1.000-1.000	<5.9%/nM	100%	84-100%	100%	91-100%

Hepcidin levels below the lower limit of detection (<0.5 nM) were imputed with a value of 0.25 nM (A) or with a value of 0.49 nM (B). 95% CI of AUC calculated with SPSS version 22.0 and 95% CI of sensitivity and specificity calculated with GraphPad Prism version 8.4.2. AUC, area under the curve; CI, confidence interval.
